# Spatial multi-omics decoding of the fracture nonunion niche: from immune–vascular–skeletal crosstalk to precision regeneration

**DOI:** 10.3389/fmed.2026.1931616

**Published:** 2026-09-04

**Authors:** Jingze Yang, Ruohui Tang, Jin Yin, Ru Sun

**Affiliations:** Department of Orthopaedics, First People’s Hospital of Kunming City & Calmette Affiliated Hospital of Kunming Medical University, Kunming, Yunnan, China

**Keywords:** fracture nonunion, mechanotransduction, osteogenic–angiogenic coupling, osteoimmunosenescence, skeletal stem and progenitor cells, spatial multi-omics, type H vessels

## Abstract

Fracture nonunion — the failure of bone to heal without surgical intervention — affects 5%–10% of all fractures and represents a substantial clinical and economic burden. Despite decades of research, the cellular and molecular mechanisms that distinguish successful repair from nonunion remain incompletely understood, largely because the fracture niche is a spatially organized, multicellular ecosystem whose crosstalk cannot be captured by bulk analyses. The recent convergence of spatial transcriptomics, single-cell multi-omics, spatial proteomics, and computational integration methods has opened a new era in which the fracture nonunion niche can be decoded at unprecedented resolution. Here we synthesize evidence from 2022 to 2025 that implicates three interdependent axes of niche dysfunction: (i) arrested macrophage polarization and chronic inflammation driven by dysregulated CSF1R, CD163, and T-cell signaling; (ii) uncoupled angiogenesis–osteogenesis despite preserved or elevated VEGF expression, mediated by impaired type H vessel formation and disrupted PDGF-BB/SLIT3/Notch signaling; and (iii) skewed skeletal stem and progenitor cell (SSPC) fate decisions toward fibrosis and adipogenesis at the expense of osteochondrogenic differentiation, governed by impaired BMP, Wnt, and IHH pathway activation. We then review how spatial multi-omics — including Visium, Xenium, MERFISH, imaging mass cytometry, and MALDI-mass spectrometry imaging — has mapped these axes *in situ*, enabling the discovery of spatially restricted therapeutic targets. Finally, we examine how these mechanistic insights are being translated into precision regeneration strategies, including bone-targeted nanoparticles that reprogram macrophage polarization, controlled-release scaffolds that restore angiogenic–osteogenic coupling, and biomaterials that reactivate endogenous SSPCs. We propose that the future of nonunion treatment lies in spatially informed, patient-specific immunomodulatory and regenerative therapies guided by multi-omic profiling of the individual fracture niche.

## 1 Introduction

Fracture healing is one of the few postnatal processes that recapitulates endochondral and intramembranous ossification, restoring bone mass and mechanical integrity without scar formation. Yet this remarkable regenerative capacity fails in approximately 5%–10% of fractures, resulting in nonunion — a painful, disabling condition that requires repeated surgical intervention and imposes annual healthcare costs exceeding billions of dollars globally (1). The incidence of nonunion is rising as populations age and metabolic comorbidities such as diabetes and osteoporosis become more prevalent (2).

The traditional view of nonunion pathogenesis emphasized mechanical instability and insufficient vascular supply. However, two decades of molecular investigation have revealed that fracture healing is orchestrated by a precisely timed sequence of cellular events — inflammation, soft callus formation, hard callus maturation, and remodeling — each governed by spatially restricted signaling among immune cells, vascular endothelial cells, and skeletal progenitors. Nonunion arises not from the failure of any single cell type but from dysregulated crosstalk within this multicellular niche (3, 4).

The major obstacle to understanding and therapeutically targeting this crosstalk has been technological: conventional transcriptomic and proteomic methods homogenize tissue, destroying the spatial context on which niche function depends. A fracture callus is a histologically zoned structure comprising hematoma, granulation tissue, cartilage, woven bone, and remodeling marrow — each zone with distinct cellular composition and signaling logic. Bulk analyses conflate these zones, obscuring the very spatial gradients that drive successful repair.

Recent advances in spatial omics have begun to address this limitation. Over the past three years, an expanding range of spatial profiling technologies has been applied to musculoskeletal tissues: spatial transcriptomics (10× Visium, Visium HD, Xenium, MERFISH, Stereo-seq), spatial proteomics (imaging mass cytometry, MIBI-TOF, GeoMx DSP), and spatial metabolomics (MALDI-MSI, DESI-MSI), together with integrated single-cell and single-nucleus sequencing (5, 6, 31). These tools make it possible, for the first time, to map gene expression, protein abundance, and metabolite distribution onto the histological architecture of the healing fracture — and to compare union vs. nonunion niches with spatial precision.

It is important to acknowledge that the application of spatial omics technologies to fracture biology remains at an early stage. The number of published studies is still limited, most reports involve small sample sizes or represent proof-of-concept demonstrations, and comprehensive spatial atlases of human fracture nonunion tissue are not yet available. Nevertheless, the studies reviewed here establish the feasibility and transformative potential of these approaches, and the pace of technological development suggests that larger-scale, multi-center studies will become feasible in the near future.

In this review, we synthesize the emerging evidence from spatial and single-cell multi-omics that defines the fracture nonunion niche as a spatially encoded failure of immune–vascular–skeletal crosstalk. We first summarize the cellular and molecular choreography of normal fracture repair, drawing on recent single-cell atlases. We then examine how spatial multi-omics technologies have been deployed to decode the fracture niche. Next, we dissect the three principal axes of niche dysfunction in nonunion: immune dysregulation, vascular–skeletal uncoupling, and SSPC fate arrest. Finally, we discuss how these mechanistic insights are being translated into precision regeneration strategies — from bone-targeted nanotherapeutics to spatially patterned biomaterials — and outline the challenges and opportunities that lie ahead.

Beyond the immune-vascular-skeletal triad, two additional biological processes fundamentally shape the fracture niche and merit explicit consideration. Mechanotransduction — the conversion of mechanical cues into biochemical signals through YAP/TAZ, Piezo1/2 ion channels, and integrin-mediated focal adhesion signaling — regulates immune cell behavior, angiogenesis, and SSPC differentiation at every stage of repair (34, 35). Likewise, aging reshapes the fracture niche through immunosenescence, skeletal stem cell dysfunction, impaired angiogenesis, and the accumulation of senescent cells secreting a pro-inflammatory SASP (36, 37). Both mechanotransduction and aging are integral to the niche pathology discussed in subsequent sections.

## 2 The cellular choreography of fracture healing: insights from single-cell atlases

### 2.1 The inflammatory phase: macrophage polarization as a fate switch

Fracture healing begins with hematoma formation and an acute inflammatory response that recruits neutrophils, monocytes, and lymphocytes to the injury site. Single-nucleus transcriptomic profiling of the fracture microenvironment has revealed that this inflammatory phase is far more complex than previously appreciated. Hachemi et al. (2024) performed multimodal single-nucleus analyses of immune cells during murine tibial fracture repair and identified four distinct macrophage subpopulations that transition through successive pro-inflammatory, pro-repair, and anti-inflammatory transcriptional states (3). The trajectory from pro-inflammatory (M1-like, expressing *Il1b*, *Tnf*, *Ccl2*) to anti-inflammatory (M2-like, expressing *Arg1*, *Cd206*, *Il10*) macrophages is essential for the resolution of inflammation and the initiation of endochondral ossification.

This trajectory is critically dependent on specific molecular mediators. Ren et al. (2023) demonstrated that the M2 scavenger receptor CD163 is required for normal fracture healing: CD163-deficient mice exhibit persistent fracture gaps, up to 74% reduction in bone volume, 40% reduction in vasculature, and significantly decreased yield torque and rigidity (7). These findings establish macrophage polarization — particularly the M1→M2 transition — as a fate switch whose failure predisposes to nonunion.

Importantly, the macrophage polarization trajectory does not occur in isolation. Murayama et al. (2024) showed that M2 macrophages promote Th2 and Treg cell expansion, which in turn enhance osteogenic differentiation of mesenchymal stromal cells (MSCs), identifying a tripartite MSC–macrophage–T-cell regulatory circuit optimized at a 1:5:10 ratio (8). This circuit, when disrupted, provides a mechanistic basis for the chronic inflammation observed in nonunion tissue.

### 2.2 Skeletal stem and progenitor cells: periosteum as the regenerative reservoir

The cellular source of bone-forming osteoblasts during repair has been the subject of longstanding debate. Single-cell and lineage-tracing studies have now resolved this question: the periosteum, not the bone marrow, harbors the primary skeletal stem and progenitor cells (SSPCs) mobilized during fracture repair. Perrin et al. (2024) generated a single-nucleus atlas of the periosteum at steady state and at days 3, 5, and 7 post-tibial fracture, identifying periosteal SSPCs marked by *Pi16* and *Ly6a/SCA1* (9). Crucially, they discovered a transient injury-induced fibrogenic cell (IIFC) population that serves as a common intermediate before SSPCs commit to either osteoblasts or chondrocytes. IIFCs are the main source of paracrine signals in the early fracture microenvironment, and their differentiation trajectories are governed by distinct gene programs involving Notch, Wnt, and circadian clock signaling.

Complementary work by Sivaraj et al. (2022) used scRNA-seq of non-hematopoietic stromal cells from post-fracture day 14 mouse bone and identified BMSC subclusters with elevated *Fabp5* and *Mmp9* expression during healing, as well as heterogeneous osteoblast lineage cells marked by *Col22a1* (10). Nakayama et al. (2022) further showed, using Sox9-CreERT2 lineage tracing and scRNA-seq of calvarial repair, that skeletal progenitors undergo a bifurcated lineage decision toward osteogenic vs. adipogenic fates — a decision regulated by Ccl9/CCR1 signaling (11).

Collectively, these single-cell atlases establish that the periosteal SSPC reservoir generates the osteoblasts and chondrocytes that build the fracture callus, that a fibrogenic intermediate state (IIFC) is a required transition, and that fate decisions are exquisitely sensitive to signals from the immune and vascular compartments.

### 2.3 Angiogenesis–osteogenesis coupling: the type H vessel paradigm

Bone formation during fracture repair is spatially and temporally coupled to angiogenesis. The discovery of type H vessels (CD31hi/Endomucinhi) by Kusumbe et al. (2014) provided a cellular basis for this coupling: these specialized capillary endothelial cells, concentrated near the growth plate and in regions of active bone formation, secrete factors that promote osteoprogenitor proliferation and differentiation (12). The molecular mediators of this coupling include PDGF-BB (secreted by pre-osteoclasts), SLIT3 (secreted by osteoblasts), and endothelial Notch/Dll4 signaling, which collectively regulate type H vessel density and geometry (13, 40).

However, the nature of angiogenic–osteogenic coupling is more nuanced than originally proposed. Schilling et al. (2024) used longitudinal intravital multiphoton microscopy to demonstrate that early vascular sprouting during calvarial healing is not directly coupled to osteoprogenitor invasion. Modulation of endothelial Notch and VEGF signaling altered vascular growth without corresponding changes in ossification (14). This finding suggests that the degree of osteo-angiogenic coupling is site-specific and that therapeutic strategies must account for anatomical context.

### 2.4 Endochondral ossification: the chondrocyte-to-osteoblast transition

A defining feature of fracture repair (as distinct from calvarial healing) is the formation of a cartilage intermediate that is subsequently replaced by bone. The cellular mechanism of this transition — whether hypertrophic chondrocytes undergo apoptosis and are replaced by invading osteoprogenitors, or whether they directly transdifferentiate into osteoblasts — has been clarified by lineage-tracing studies, which support a direct chondrocyte-to-osteoblast transdifferentiation pathway for a subset of callus chondrocytes. More recently, Tachibana et al. (2024) demonstrated that the transcription factor KLF15 regulates endochondral ossification during fracture healing through a TGF-β–SMAD3–SOX9 axis that operates independently of IHH signaling, revealing additional layers of transcriptional control over chondrocyte maturation (45).

Complementary studies have provided further insight into the spatial and molecular regulation of chondrocyte maturation. Bian et al. (2023) applied spatial transcriptomics to mineralized mouse knee joints and identified a requirement for ADGRG6 in maintaining resting-zone chondrocyte homeostasis (15). Related work has further characterized resting-zone subpopulations in human growth plates (32). In the context of fracture repair, Ma et al. (2022) demonstrated that PTH 1–34 promotes endochondral ossification through CREB-mediated activation of IHH signaling (46).

At the human level, To et al. (2024) generated a multiomic atlas of human early skeletal development using paired snRNA-seq, snATAC-seq, and spatial transcriptomics (155-plex *in situ* sequencing and Visium), revealing regionally distinct osteoprogenitor trajectories and regulatory networks governing intramembranous vs. endochondral ossification (17, 33). These studies provide a valuable molecular foundation for investigating how the normal endochondral sequence may be disrupted in nonunion.

## 3 Spatial multi-omics: technologies enabling niche decoding

### 3.1 The technology landscape

The spatial multi-omics toolkit applicable to bone has expanded dramatically since 2022. Two comprehensive 2025 reviews in Bone Research provide authoritative surveys of this landscape (5, 6). The principal platforms, summarized in Table 1, fall into three categories:

**Table 1:** Spatial multi-omics platforms applicable to bone and fracture research.

Platform	Category	Resolution	Multiplex capacity	Bone application highlights
10× Visium	Sequencing-based ST	55 µm spots	Transcriptome-wide	Fracture callus zonation; FFPE-compatible
Visium HD	Sequencing-based ST	2 µm bins	Transcriptome-wide	Near-single-cell spatial mapping
Stereo-seq	Sequencing-based ST	500 nm spots	Transcriptome-wide	Ultra-high-resolution spatial profiling
Xenium (10×)	Imaging-based ST	Subcellular	∼400-plex	Osteochondral junction; IF co-detection
MERFISH (Vizgen)	Imaging-based ST	Subcellular	Up to 500-plex	Single-cell spatial gene expression
CosMx SMI	Imaging-based ST	Subcellular	Up to 1,000-plex	High-plex spatial profiling
Imaging Mass Cytometry	Spatial proteomics	1 µm	40–100 + markers	Immune cell spatial mapping
MIBI-TOF	Spatial proteomics	1 µm	40–100 + markers	Protein-level niche characterization
GeoMx DSP	Spatial proteomics/transcriptomics	ROI-based	Proteome/transcriptome-wide	Region-of-interest profiling from FFPE
MALDI-MSI	Spatial metabolomics	10–50 µm	Hundreds of metabolites	Metabolite/lipid distribution mapping

Sequencing-based spatial transcriptomics captures polyadenylated RNA on spatially barcoded arrays. 10× Visium (55 µm spot diameter) has been the most widely adopted platform for bone applications, with Visium HD (2 µm binned resolution) now enabling near-single-cell spatial resolution. Stereo-seq (500 nm spots) and Slide-seqV2 (10 µm beads) offer higher resolution alternatives. These platforms are compatible with FFPE tissue following decalcification — a critical requirement for mineralized bone. Miao et al. (2025) recently published an optimized protocol using Morse's solution for rapid decalcification (<24 h) combined with hydrogel-coated slides, achieving >10-fold improvement in gene detection (∼5,600 genes per spot) compared with conventional EDTA-based methods (47).

Imaging-based spatial transcriptomics uses fluorescent probes to detect transcripts *in situ*. Xenium (10× Genomics, ∼400-plex), MERFISH (Vizgen, up to 500-plex), and CosMx (NanoString, up to 1,000-plex) achieve subcellular resolution and can be combined with immunofluorescence for protein co-detection. These platforms are particularly valuable for resolving fine anatomical structures such as the osteochondral junction and the type H vessel microenvironment.

Spatial proteomics and metabolomics complement transcriptomic data by measuring the functional effectors of niche signaling. Imaging mass cytometry (IMC) and MIBI-TOF use metal-tagged antibodies to detect 40–100+ proteins simultaneously with 1 µm resolution. GeoMx Digital Spatial Profiler (DSP) enables region-of-interest-based proteomic or transcriptomic profiling from FFPE sections. For metabolites, MALDI-mass spectrometry imaging (MALDI-MSI) maps hundreds of metabolites, lipids, and small signaling molecules directly onto tissue architecture. A 2024 review in Bone Research highlighted that spatial proteomics and metabolomics, while less mature than spatial transcriptomics, are poised to play a crucial role in musculoskeletal research by capturing the functional effectors of niche signaling that transcript-level measurements alone cannot resolve (52).

### 3.2 Applications to bone and fracture

Jiang et al. (2024) used Visium CytAssist to spatially characterize the murine femoral fracture callus, validating known zonation markers — hard callus (Dmp1, Sost) and soft callus (Acan, Col2a1) — and identifying downregulation of bone matrix regulators (Bglap, SPARC, Tnc) in a pathological (breast cancer metastasis) fracture model (18).

The most clinically relevant application to date comes from Rios et al. (2024), who applied spatial transcriptomics to both murine Nf1-deficient fracture callus and human NF1 pseudarthrosis (nonunion) tissue (19). This study revealed absent BMP pathway activation (confirmed by phospho-SMAD1/5/8 immunohistochemistry) and increased MAPK signaling specifically at the fibrocartilaginous–osseous junction — precisely the zone where the chondrocyte-to-osteoblast transition occurs. This spatially resolved finding would have been impossible to detect by bulk RNA-seq and nominates BMP2 as a junction-specific therapeutic target.

Mathavan et al. (2025) introduced a “mechanomics” platform that integrates time-lapsed *in vivo* micro-CT, Visium spatial transcriptomics, and micro-finite element analysis to map gene expression as a function of local mechanical strain within fracture sites (20). They identified strain-dependent regulation of *Bglap*, *Sost*, *Wnt7b*, and *Il11*, providing a direct link between mechanical loading and spatially defined transcriptomic responses.

Wang et al. (2025) integrated spatial transcriptomics (10× Visium) with single-cell transcriptomics to characterize the mouse femoral fracture healing process at three time points (Days 0, 5, and 15 post-fracture), identifying 27 unique spatial clusters and mapping the critical transformation of mesenchymal progenitor cells (MPCs) into regenerative MPCs (rMPCs) that recruit macrophages to the fracture line during early healing (16). This study also revealed spatiotemporal shifts in gene expression from bone marrow (Day 0) to periosteal regions (Day 5) to the fracture callus (Day 15), providing a comprehensive spatial framework for understanding the cellular dynamics of bone regeneration.

### 3.3 Data integration and computational challenges

Beyond the bone-specific challenges noted above, spatial multi-omics studies of complex niches face well-recognized computational hurdles that deserve explicit consideration. Integrating data across multiple modalities (transcriptomics, proteomics, metabolomics) in a spatially resolved context requires sophisticated computational frameworks that can align datasets with inherently different spatial resolutions, dynamic ranges, and noise profiles (38). Normalizing data across patient samples to account for inter-individual heterogeneity — particularly in clinically heterogeneous conditions such as fracture nonunion — remains an unsolved challenge. Confounding factors such as patient age, comorbidities (e.g., diabetes, smoking, osteoporosis), fracture anatomical location, fixation method, and prior treatment history are known to influence nonunion biology, yet existing spatial omics studies have not systematically accounted for these variables. Furthermore, functional validation of computationally predicted crosstalk mechanisms — through genetic perturbation, pharmacological inhibition, or *ex vivo* niche reconstitution — remains a critical bottleneck. Without such validation, spatially co-localized expression patterns cannot be distinguished from functionally meaningful ligand-receptor interactions. Addressing these gaps will require multi-center consortia, standardized sample collection and data processing pipelines, and close integration of computational predictions with functional validation in physiologically relevant models (21, 38).

The integration of spatial transcriptomic data with scRNA-seq reference atlases, spatial proteomics, and histology is a major computational frontier. Yip et al. (2025) reviewed the emerging suite of computational tools for spatial multi-omics integration in the bone marrow microenvironment (21). Key challenges specific to bone include: (i) decalcification-induced RNA degradation that varies spatially depending on tissue mineralization; (ii) the need to segment and annotate distinct tissue zones (hematoma, cartilage, woven bone, marrow) before differential expression analysis; (iii) integration of 2D spatial data with 3D tissue architecture; and (iv) the relatively small number of spatial omics studies in bone, limiting the statistical power of meta-analyses. Beyond these bone-specific considerations, the broader spatial omics computational ecosystem has matured rapidly, with specialized tools now available for spatially aware cell-cell communication inference (COMMOT, CellNEST), spatial domain detection (SpaGCN, BayesSpace), and multi-sample integration (NicheCompass) (48).

## 4 Immune–vascular–skeletal crosstalk in the fracture nonunion niche

### 4.1 Immune dysregulation: chronic inflammation and arrested macrophage polarization

The earliest cellular hallmark of fracture nonunion is the failure to resolve acute inflammation. Song et al. (2024) provided a comprehensive review of immunomodulation in bone nonunion, cataloging roles for macrophages, T cells, neutrophils, and fibroblast growth factors in controlling bone repair (1). The core defect appears to lie at the M1 → M2 macrophage polarization checkpoint.

In normal healing, pro-inflammatory macrophages clear debris and secrete cytokines that recruit SSPCs, then transition to an M2 phenotype that secretes osteogenic factors (BMP2, TGF-β, VEGF) and promotes angiogenesis. In nonunion, this transition is arrested, resulting in sustained M1-dominant inflammation. Hachemi et al. (2024) demonstrated that pharmacological depletion of pathological macrophages using the CSF1R inhibitor Pexidartinib improved bone healing in a murine musculoskeletal trauma model (3).

Coates et al. (2022) performed single-cell RNA-seq of intramedullary canal tissue from femoral nonunion patients (*n* = 5) vs. native bone controls (*n* = 4) and identified 23 distinct cell clusters, with nonunions showing increased monocytes and CD14+ dendritic cells and decreased T cells, myelocytes, and promyelocytes (22). Osteoblast, chondrocyte, and tissue stem cell proportions were also altered, suggesting that immune dysregulation propagates to the skeletal compartment.

Epigenetic mechanisms may underlie the failure of macrophage polarization. Xiao et al. (2023) identified DNA methylation-mediated suppression of Rbpjk (a Notch pathway transcriptional effector) as a protective mechanism against inflammation-induced fracture nonunion (23). This finding links environmental inflammatory insults to heritable changes in Notch signaling capacity and suggests that epigenetic biomarkers could stratify nonunion risk.

### 4.2 Vascular–skeletal uncoupling: the vascularization paradox

The relationship between vascular supply and fracture healing is more complex than the traditional “ischemia causes nonunion” model. Menger et al. (2022) coined the term “vascularization paradox of non-union formation” based on evidence that nonunion callus tissue is often densely vascularized and that VEGF is sufficiently (or even over-) expressed at the fracture site (4). The critical defect appears to lie not in vascular quantity but in vascular quality and function — specifically, the failure to form type H vessels that couple angiogenesis to osteogenesis.

The molecular basis for this uncoupling involves disrupted PDGF-BB/SLIT3/Notch signaling. Type H vessel endothelial cells depend on PDGF-BB (secreted by pre-osteoclasts and M2 macrophages) and SLIT3 (secreted by osteoblasts) for their maintenance, and they signal back to perivascular osteoprogenitors via Notch ligands (12, 13). In the nonunion microenvironment, the M1-dominant immune milieu reduces PDGF-BB availability, and SSPC dysfunction (see Section 4.3) reduces SLIT3 production — creating a feed-forward loop in which failed immune resolution impairs type H vessel maturation, which in turn deprives osteoprogenitors of necessary angiocrine signals.

The site-specificity of this coupling is underscored by the 2024 demonstration that angiogenesis and osteogenesis can be mechanistically uncoupled in calvarial bone (14), highlighting the need for spatial context in therapeutic design.

### 4.3 SSPC fate arrest: fibrosis and adipogenesis at the Expense of osteogenesis

The third axis of niche dysfunction involves the fate decisions of skeletal stem and progenitor cells. In normal healing, periosteal SSPCs pass through a fibrogenic intermediate (IIFC) before committing to osteochondrogenic differentiation (9). In nonunion, this trajectory is disrupted at two points: (i) SSPCs fail to exit the fibrogenic state, resulting in fibrous nonunion; or (ii) SSPCs are diverted toward adipogenesis, resulting in atrophic nonunion with fatty marrow infiltration.

Nakayama et al. (2022) identified Ccl9/CCR1 signaling as a regulator of the osteogenic-vs.-adipogenic fate decision in skeletal progenitors, with Ccl9 promoting adipogenesis and pharmacological Ccl9 blockade improving bone regeneration (11). The spatial context of this decision is critical: SSPCs located near the periosteal surface in an M2-dominant microenvironment receive BMP and Wnt signals that promote osteogenesis (43), whereas SSPCs exposed to chronic M1-derived cytokines (TNF-α, IL-1β) activate adipogenic programs.

Aging compounds this vulnerability. Ambrosi et al. (2025) demonstrated that skeletal stem cells from aged mice have diminished bone- and cartilage-forming potential and instead produce pro-inflammatory, pro-resorptive stromal lineages, and that combinatorial anti-CSF1 + BMP2 treatment can reactivate aged SSCs and restore youthful healing (24). This finding suggests that age-related SSPC dysfunction is pharmacologically reversible — a principle with direct implications for elderly patients with nonunion. Beyond SSPC-intrinsic dysfunction, aging also acts through the immune compartment: immunosenescence drives a chronic, low-grade inflammatory state characterized by SASP secretion from senescent osteocytes, macrophages, and stromal cells, which can induce paracrine senescence in otherwise functional SSPCs (36). The concept of “osteoimmunosenescence” has emerged as a unifying framework describing how age-related immune remodeling — including thymic involution, T-cell repertoire contraction, and macrophage dysfunction — converges upon the skeletal system to impair bone repair (49). Zou et al. (2024) identified grancalcin, an age-related macrophage-secreted factor, as a direct inducer of SSPC senescence during fracture healing, providing a molecular link between aging macrophages and impaired bone regeneration (36). King et al. (2025) demonstrated that aged periosteal SSPCs exhibit reduced proliferation, diminished chondrogenic gene expression, and increased senescence signatures, accompanied by elevated Cxcl9-mediated CD8+ T cell recruitment to the fracture site (37). These findings collectively indicate that aging creates a self-amplifying 'senescent niche' in which SASP factors from multiple cell types reinforce SSPC dysfunction — a feed-forward loop that may be pharmacologically targetable through senolytics or immunomodulatory interventions (24, 42).

### 4.4 The spatial architecture of the nonunion niche

Mechanotransduction integrates mechanical cues into the molecular signaling networks described above. The mechanosensitive transcriptional co-activators YAP and TAZ function as central hubs that convert matrix stiffness, fluid shear, and cyclic strain into osteogenic gene programs through interactions with RUNX2 and β-catenin (34). In the context of fracture repair, mechanical loading regulates immune cell behavior, angiogenic sprouting, and SSPC lineage commitment. Mehl et al. (2024) demonstrated that external mechanical stability — rather than endothelial YAP/TAZ signaling *per se* — is the dominant regulator of hematoma vascularization during early bone healing, highlighting the primacy of biomechanical context (35). Conversely, the mechanomics platform of Mathavan et al. (2025) showed that local mechanical strain directly modulates the expression of osteogenic (Bglap, Spp1) and mechanosensitive (Il11, Wnt7b, Sost) genes within the fracture callus (20). These findings suggest that nonunion may arise not only from failed biochemical signaling but also from aberrant mechanotransduction — a dimension that spatial multi-omics is uniquely positioned to interrogate by correlating local strain fields with transcriptomic responses.

The three axes described above — immune dysregulation, vascular–skeletal uncoupling, and SSPC fate arrest — are not independent. They are spatially organized into a dysfunctional niche whose architecture is only now being mapped by spatial multi-omics. The key spatial compartments of the nonunion niche, informed by recent spatial transcriptomic data (16, 19, 20), include:

(1) The central hematoma/fibrotic zone: enriched in M1 macrophages, neutrophils, and activated myofibroblasts; high TNF-α and IL-1β; low BMP and TGF-*β* signaling.
(2) The fibrocartilaginous–osseous junction: the critical transition zone where chondrocytes should convert to osteoblasts but where, in nonunion, BMP–SMAD signaling is absent and MAPK signaling is elevated (19).
(3) The periosteal niche: where SSPCs normally reside but where, in nonunion, the IIFC state persists and osteogenic commitment fails.
(4) The perivascular niche: where type H vessels should couple to osteoprogenitors but where PDGF-BB/SLIT3/Notch signaling is disrupted.

Each of these compartments represents a spatially defined therapeutic target that could be addressed by precision delivery systems. The major immune–vascular–skeletal crosstalk axes underlying the fracture nonunion niche are summarized in Table 2.

**Table 2:** Immune–vascular–skeletal crosstalk axes in the fracture nonunion niche.

Niche axis	Normal healing	Nonunion defect	Key molecular mediators	References
Immune regulation (Macrophage polarization)	M1 → M2 macrophage transition; resolution of acute inflammation; Treg expansion promotes osteogenesis	Arrested M1-dominant inflammation; failed M2 polarization; sustained TNF-α and IL-1β	CSF1R, CD163, TNF-α, IL-1β, IL-10, TGF-β, BMP2, Rbpjk (Notch effector)	(1, 3, 7, 8, 22, 23)
Angiogenesis– osteogenesis coupling (Type H vessels)	Type H vessel (CD31hi/Endomucinhi) formation; PDGF-BB/SLIT3/Notch-mediated angiocrine signaling	Preserved VEGF but failed type H vessel maturation; vascular–skeletal uncoupling	PDGF-BB, SLIT3, Notch/Dll4, VEGF	(4, 12–14)
SSPC fate determination (Periosteal niche)	Periosteal SSPCs → IIFC → osteochondrogenic differentiation under BMP/Wnt/IHH signaling	Fibrogenic arrest (fibrous nonunion) or adipogenic diversion (atrophic nonunion); failed osteogenic commitment	BMP2, Wnt/β-catenin, IHH, Ccl9/CCR1, Notch, Pi16, Ly6a/SCA1	(9–11, 24)

## 5 From mechanistic insight to precision regeneration

### 5.1 Bone-targeted nanoparticles for macrophage reprogramming

The recognition that macrophage polarization is a master regulator of the fracture niche has spurred the development of bone-targeted nanotherapeutics. Xiao et al. (2024) developed PSMA-b-PS nanoparticles functionalized with a TRAP-binding peptide (TBP) for bone surface targeting, loaded with the Wnt/β-catenin agonist AR28 (25). In a murine fracture model, TBP-NP/AR28 achieved approximately 60% uptake by macrophages at the fracture site, shifted the M2/M1 ratio, and yielded approximately 4-fold greater torsional rigidity compared with controls. Critically, the therapeutic effect was mediated primarily through macrophage polarization — not direct osteoblast activation — establishing macrophage reprogramming as a viable and targetable therapeutic axis.

Griffin et al. (2025) used a Design of Experiments approach to identify optimal immunomodulatory factors (IL-10 and IL-4) and loaded them into polymeric microparticles for controlled release, demonstrating concurrent MSC osteogenic differentiation and macrophage M2 polarization under chronic inflammatory conditions (26). In a complementary approach, Qin et al. (2025) developed a ZIF-8-based hydrogen sulfide (H₂S) delivery system with a bone-targeting peptide (SDSSD) that selectively releases H₂S in bone tissue, reprogramming macrophage metabolism by suppressing succinate accumulation and mitochondrial ROS, thereby promoting M2 polarization and osteoblast-osteoclast coupling (44).

### 5.2 Controlled-release scaffolds for angiogenic–osteogenic recoupling

Restoring type H vessel-mediated coupling between angiogenesis and osteogenesis requires spatiotemporally controlled delivery of the relevant molecular signals. Zhang et al. (2024) catalogued delivery platforms including polyurethane/microsphere composites (burst + sustained BMP-2 release), silk-fibroin/calcium phosphate scaffolds, PLGA double-walled microspheres (sequential FGF-2 then BMP-2), PLLA nanosheets, bioactive glass, and GelMA hydrogels (27). The translational gap highlighted by this review is that most preclinical models use acute critically-sized defects rather than chronic nonunion models, which fail to recapitulate the inflammatory and fibrotic microenvironment of established nonunion.

Marin et al. (2025) outlined the most forward-looking strategies: controlled-release scaffolds that co-deliver angiogenic (VEGF, PDGF-BB, SLIT3) and osteogenic (BMP-2, BMP-6) factors in a spatially patterned manner, immunomodulatory materials that actively reprogram the local immune environment, and stem cell-derived exosomes as cell-free alternatives (28). Luo et al. (2025) reviewed biomaterials that target type H vessel formation through modulation of VEGF, HIF-1*α*, PDGF-BB, SLIT3, and Notch signaling axes, highlighting that the next generation of bone biomaterials should incorporate functional verification capacity for type H vessel-mediated angiogenic-osteogenic coupling (50).

### 5.3 Reactivating endogenous skeletal stem cells

The discovery that SSPCs persist at nonunion sites but are arrested in fibrogenic or adipogenic states has motivated strategies to reactivate their osteochondrogenic potential. The Ambrosi et al. (2025) demonstration that anti-CSF1 + BMP2 combination therapy reactivates aged SSCs establishes a proof-of-concept that endogenous progenitors can be pharmacologically reprogrammed (24). The spatial transcriptomic identification of the fibrocartilaginous–osseous junction as the critical BMP-responsive zone in human nonunion tissue (19) provides an anatomical target for such reactivation.

### 5.4 Clinical translation: orthobiologics and personalized approaches

The clinical armamentarium for nonunion treatment is evolving from generic biologics toward precision approaches. Kiwan et al. (2025) systematically reviewed orthobiologics for nonunion, finding that BMP-2 products show the strongest evidence for tibial nonunion (both septic and aseptic), BMP-7 evidence is inconclusive, and concentrated bone marrow aspirate achieves 75%–90% success rates for tibial nonunions when combined with appropriate scaffolds (29).

Giannoudis et al. (2024) concluded that the heterogeneity of nonunion — in terms of biology, mechanical stability, and host factors — demands personalized treatment protocols that match specific biologics to patient and fracture subtypes (30). A parallel 2024 clinical consensus statement emphasized that risk stratification for nonunion should incorporate patient-specific biological factors — including age, metabolic status, and inflammatory burden — alongside mechanical considerations, and that future clinical algorithms should integrate molecular biomarkers of the three niche dysfunction axes described in this review (51). The logical next step is to incorporate spatial multi-omic profiling of individual nonunion biopsies into treatment planning — analogous to tumor molecular profiling in oncology — to guide selection of immunomodulatory, angiogenic, or osteogenic therapies based on the patient's specific niche defect. The principal precision regeneration strategies discussed above are summarized in Table 3.

**Table 3:** Precision regeneration strategies for fracture nonunion.

Strategy	Mechanism	Key preclinical/clinical findings	Development stage	References
Bone-targeted nanoparticles	TBP-functionalized NPs deliver Wnt/β-catenin agonist (AR28) to fracture-site macrophages; reprogram M1 → M2 polarization	∼60% macrophage uptake at fracture site; M2/M1 ratio shift; ∼4-fold greater torsional rigidity vs. controls in murine model	Preclinical (*in vivo*)	(25, 26)
Controlled-release scaffolds	Spatiotemporally controlled co-delivery of angiogenic (VEGF, PDGF-BB, SLIT3) and osteogenic (BMP-2, BMP-6) factors; immunomodulatory biomaterials	Sequential FGF-2 → BMP-2 release; PLGA double-walled microspheres; GelMA hydrogels; stem cell-derived exosomes as cell-free alternatives	Preclinical (*in vivo*)	(27, 28)
SSPC reactivation	Anti-CSF1 + BMP2 combination therapy; pharmacological reprogramming of aged or suppressed skeletal stem/progenitor cells	Reactivates aged SSCs; restores youthful bone- and cartilage-forming capacity; targets fibrocartilaginous–osseous junction	Preclinical (*in vivo*)	(24)
Orthobiologics (clinical translation)	BMP-2, BMP-7, concentrated bone marrow aspirate (cBMA); patient-specific niche-informed biologic selection	BMP-2: strongest evidence for tibial nonunion (septic and aseptic); cBMA: 75%–90% success in tibial nonunions with appropriate scaffolds; BMP-7: inconclusive	Clinical/approved	(29, 30)

## 6 Discussion

The evidence synthesized in this review establishes that fracture nonunion is fundamentally a disorder of spatially encoded intercellular communication. The three axes of niche dysfunction described above — arrested macrophage polarization, uncoupled angiogenesis–osteogenesis, and skewed SSPC fate decisions — are mechanistically interdependent, forming a feed-forward circuit in which the failure of any one axis destabilizes the others. For instance, the M1-dominant immune milieu reduces PDGF-BB availability from pre-osteoclasts and macrophages while SSPC dysfunction diminishes SLIT3 production, together impairing type H vessel maturation; the resulting deficit in angiocrine signals (Notch ligands, Wnt factors) further suppresses osteoprogenitor commitment, perpetuating fibrous or adipogenic nonunion. This interdependence carries a direct therapeutic implication: strategies targeting a single niche axis in isolation may be insufficient, and the most effective interventions are likely to be those that restore coordinated immune–vascular–skeletal crosstalk, as exemplified by the combinatorial anti-CSF1 + BMP2 approach (24).

Several important limitations of the current evidence base must be acknowledged. First, the vast majority of spatial omics studies in bone have been conducted in murine models; human nonunion spatial atlases remain restricted to a single proof-of-concept study in NF1 pseudarthrosis (19) and one scRNA-seq study (22). Second, because spatial profiling generally requires destructive tissue sampling, existing studies provide cross-sectional snapshots rather than longitudinal measurements from the same healing tissue, which limits causal inference about which niche defects initiate the nonunion trajectory vs. which arise as secondary or compensatory responses. Third, the decalcification protocols required for mineralized bone tissue introduce RNA degradation artifacts that vary spatially with the degree of tissue mineralization, complicating quantitative cross-zone comparisons. Fourth, nonunion is a clinically heterogeneous condition encompassing atrophic, hypertrophic, oligotrophic, infected, osteoporotic, and diabetic subtypes; niche defects identified in one subtype may not extend to others. Fifth, the majority of preclinical therapeutic studies reviewed here were performed in acute critically sized defect models rather than established nonunion models, which fail to recapitulate the chronic inflammatory and fibrotic microenvironment that characterizes clinical nonunion (27). Sixth, mechanotransduction and aging — two processes that fundamentally regulate fracture repair — have not yet been systematically interrogated by spatial multi-omic approaches in the context of nonunion, representing important gaps in the current evidence base. Despite these limitations, the mechanistic framework of immune–vascular–skeletal crosstalk, now spatially resolved by multi-omic technologies, provides a rational foundation for the precision regeneration strategies discussed below. Seventh, the practical barriers to translating spatial multi-omics profiling into routine clinical care are substantial. Current spatial omics assays remain costly (approximately $3,000 per slide for the least expensive platforms), have long turnaround times that are incompatible with urgent surgical decision-making, require specialized bioinformatics infrastructure and expertise, and — critically — lack standardized, regulatory-grade analysis pipelines suitable for clinical use (39). The field also faces fundamental trade-offs between spatial resolution, molecular coverage, and throughput that complicate clinical workflow design. Addressing these barriers will require concerted efforts in technology development (to reduce cost and turnaround time), computational standardization (to enable cross-platform reproducibility), and regulatory engagement (to establish validation frameworks for spatially resolved biomarkers) (39).

### 6.1 Spatially resolved multi-omic atlases of human fracture nonunion

The most pressing need is the generation of comprehensive spatial multi-omic atlases of human fracture nonunion tissue. The proof-of-concept study by Rios et al. (2024) in NF1 pseudarthrosis and the human nonunion scRNA-seq study from 2022 provide important foundations, but larger cohorts spanning diverse nonunion etiologies (atrophic, hypertrophic, infected, osteoporotic, diabetic) are needed. These atlases should integrate spatial transcriptomics, spatial proteomics (IMC or MIBI-TOF), and spatial metabolomics (MALDI-MSI) on serial sections from the same biopsies, enabling multi-modal spatial registration.

### 6.2 Longitudinal *in vivo* spatial profiling

Because spatial omics generally requires destructive tissue sampling, existing studies provide cross-sectional snapshots rather than longitudinal measurements from the same healing tissue. Understanding the dynamics of niche dysfunction — when and how a healing niche diverges toward nonunion — requires longitudinal profiling. The mechanomics platform of Mathavan et al. (2025) provides one model for how longitudinal imaging can be integrated with terminal spatial profiling. Non-invasive molecular imaging modalities (e.g., PET reporters of macrophage polarization or BMP pathway activation) could enable within-subject monitoring of niche state over time.

### 6.3 Spatially informed computational models

Complementary computational approaches include the micro-multiphysics agent-based (micro-MPA) model developed by Kendall et al. (2023), which integrates cellular behavior, vascularization, cytokine signaling, and the local mechanical environment to simulate bone regeneration following osteotomy (53). Although this model was not originally parameterized using spatial omics data, future integration with spatially resolved transcriptomic and proteomic measurements could substantially improve its biological specificity and patient-level predictive capacity. In parallel, the mechanomics platform of Mathavan et al. (2025) — which couples time-lapsed micro-CT, micro-finite element analysis, and spatial transcriptomics — has already demonstrated that local mechanical strain fields can be correlated with spatially defined transcriptomic responses, providing a blueprint for how omics data can inform and constrain physics-based models of fracture healing (20).

The integration of spatial multi-omic data with computational models of cell–cell communication (e.g., CellChat, NicheNet, COMMOT, and newer graph neural network-based frameworks) trained on spatial coordinates will enable in silico perturbation of specific niche components. These models could predict, for example, whether a given patient's nonunion is most likely to respond to macrophage reprogramming, type H vessel restoration, or SSPC reactivation — guiding personalized therapy selection. A comprehensive 2024 review by Armingol et al. catalogued the rapidly diversifying ecosystem of cell-cell communication inference tools and highlighted the critical importance of spatially aware methods that account for tissue architecture (41).

### 6.4 Smart biomaterials with closed-loop control

The convergence of spatial omics with biomaterials engineering points toward “smart” scaffolds that sense the local niche environment and respond adaptively. For instance, a scaffold could release an M2-polarizing cytokine in response to elevated local TNF-α (indicating persistent M1 inflammation), then switch to releasing BMP-2 once inflammation resolves. Such closed-loop control would recapitulate the natural sequence of niche signals that is absent in nonunion.

### 6.5 Clinical trial design

Randomized controlled trials of precision nonunion therapies should incorporate pre-treatment spatial omic profiling of the nonunion niche and stratify patients by their dominant niche defect (inflammatory, vascular, or skeletal progenitor dysfunction). Biomarker-defined subgroups are more likely to demonstrate treatment efficacy than unselected nonunion populations, and a “basket trial” design — in which patients with the same niche defect but different fracture anatomies receive the same targeted therapy — may accelerate clinical translation.

## 7 Conclusion

The fracture nonunion niche is a spatially organized ecosystem in which immune, vascular, and skeletal cells engage in continuous bidirectional crosstalk. The failure of this crosstalk — through arrested macrophage polarization, uncoupled angiogenesis–osteogenesis, and skewed SSPC fate decisions — defines the nonunion state. Spatial multi-omics technologies, applied for the first time to bone fracture tissue between 2022 and 2025, have mapped these dysfunctions onto the histological architecture of the nonunion niche, revealing anatomically specific therapeutic targets that were invisible to bulk analyses.

The translational horizon is precision regeneration: bone-targeted nanoparticles that reprogram macrophages at the fracture site, spatially patterned scaffolds that restore type H vessel-mediated angiogenic–osteogenic coupling, and pharmacological reactivation of endogenous SSPCs whose osteogenic potential has been suppressed but not extinguished. Realizing this vision will require spatially resolved multi-omic atlases of human nonunion, longitudinal *in vivo* profiling platforms, and clinical trials that use niche biomarkers to match patients to mechanism-targeted therapies. The tools to decode the nonunion niche now exist; the next challenge is to translate this molecular map into regenerative therapies that restore the natural healing competence of bone.

## Abbreviations

ADGRG6: adhesion G protein-coupled receptor G6
AR28: Wnt/β-catenin agonist AR28
BMP: bone morphogenetic protein
BMSC: bone marrow stromal cell
cBMA: concentrated bone marrow aspirate
CCR1: C-C chemokine receptor 1
CD: cluster of differentiation
CD163: cluster of differentiation 163
CSF1: colony-stimulating factor 1
CSF1R: colony-stimulating factor 1 receptor
CT: computed tomography
DESI-MSI: desorption electrospray ionization mass spectrometry imaging
Dll4: Delta-like ligand 4
DSP: Digital Spatial Profiler
FFPE: formalin-fixed paraffin-embedded
FGF-2: fibroblast growth factor-2
GelMA: gelatin methacryloyl
GeoMx DSP: GeoMx Digital Spatial Profiler
GPR126: G protein-coupled receptor 126
IHH: Indian hedgehog
IIFC: injury-induced fibrogenic cell
IL: interleukin
IL-1β: interleukin-1 beta
IL-4: interleukin-4
IL-10: interleukin-10
IMC: imaging mass cytometry
M1: classically activated macrophage phenotype
M2: alternatively activated macrophage phenotype
MALDI-MSI: matrix-assisted laser desorption/ionization mass spectrometry imaging
MAPK: mitogen-activated protein kinase
MERFISH: multiplexed error-robust fluorescence *in situ* hybridization
MIBI-TOF: multiplexed ion beam imaging by time-of-flight
MSC: mesenchymal stromal cell
NF1: neurofibromatosis type 1
NP: nanoparticle
ORS: Orthopaedic Research Society
PDGF-BB: platelet-derived growth factor-BB
PET: positron emission tomography
PLGA: poly(lactic-co-glycolic acid)
PLLA: poly-L-lactic acid
PSMA-b-PS: poly(styrene-alt-maleic anhydride)-block-polystyrene
RAG: retrieval-augmented generation
RNA-seq: RNA sequencing
ROI: region of interest
scRNA-seq: single-cell RNA sequencing
SLIT3: slit guidance ligand 3
SMAD: small mothers against decapentaplegic
SMI: spatial molecular imaging
snATAC-seq: single-nucleus assay for transposase-accessible chromatin sequencing
snRNA-seq: single-nucleus RNA sequencing
SSPC: skeletal stem and progenitor cell
ST: spatial transcriptomics
TBP: TRAP-binding peptide
TGF-β: transforming growth factor-beta
Th2: T helper 2 cell
TNF-α: tumor necrosis factor-alpha
TRAP: tartrate-resistant acid phosphatase
Treg: regulatory T cell
VEGF: vascular endothelial growth factor
Wnt: Wingless/Int signaling pathway.
